# Rac1 plays an essential role in axon growth and guidance and in neuronal survival in the central and peripheral nervous systems

**DOI:** 10.1186/s13064-015-0049-3

**Published:** 2015-09-23

**Authors:** Zhong L. Hua, Francesco E. Emiliani, Jeremy Nathans

**Affiliations:** Department of Molecular Biology and Genetics, Johns Hopkins University School of Medicine, Baltimore, MD 21205 USA; Department of Neuroscience, Johns Hopkins University School of Medicine, Baltimore, MD 21205 USA; Department of Ophthalmology, Johns Hopkins University School of Medicine, Baltimore, MD 21205 USA; Howard Hughes Medical Institute, Johns Hopkins University School of Medicine, Baltimore, MD 21205 USA; Present address: Rockefeller University, 1230 York Avenue, New York, NY 10065 USA

**Keywords:** Planar cell polarity, Brain, Spinal cord, Retina, Motor neuron, Sensory neuron

## Abstract

**Background:**

Rac1 is a critical regulator of cytoskeletal dynamics in multiple cell types. In the nervous system, it has been implicated in the control of cell proliferation, neuronal migration, and axon development.

**Results:**

To systematically investigate the role of Rac1 in axon growth and guidance in the developing nervous system, we have examined the phenotypes associated with deleting *Rac1* in the embryonic mouse forebrain, in cranial and spinal motor neurons, in cranial sensory and dorsal root ganglion neurons, and in the retina. We observe a widespread requirement for *Rac1* in axon growth and guidance and a cell-autonomous defect in axon growth in *Rac1*^*−/−*^ motor neurons in culture. Neuronal death, presumably a secondary consequence of the axon growth and/or guidance defects, was observed in multiple locations. Following deletion of *Rac1* in the forebrain, thalamocortical axons were misrouted inferiorly, with the majority projecting to the contralateral thalamus and a minority projecting ipsilaterally to the ventral cortex, a pattern of misrouting that is indistinguishable from the pattern previously observed in *Frizzled3*^*−/−*^ and *Celsr3*^*−/−*^ forebrains. In the limbs, motor-neuron-specific deletion of *Rac1* produced a distinctive stalling of axons within the dorsal nerve of the hindlimb but a much milder loss of axons in the ventral hindlimb and forelimb nerves, a pattern that is virtually identical to the one previously observed in *Frizzled3*^*−/−*^ limbs.

**Conclusions:**

The similarities in axon growth and guidance phenotypes caused by *Rac1*, *Frizzled3*, and *Celsr3* loss-of-function mutations suggest a mechanistic connection between tissue polarity/planar cell polarity signaling and Rac1-dependent cytoskeletal regulation.

## Background

Small GTPases act as molecular toggle switches, cycling between active GTP-bound and inactive GDP-bound states to regulate a wide variety of intracellular processes in response to extracellular signals [1]. Rac1, a widely expressed member of the Rho GTPase family, controls actin dynamics and plays an essential role in cell proliferation and migration in multiple contexts [2].

In mice, constitutive deletion of *Rac1* leads to early embryonic lethality [3]. However, selective deletion of *Rac1* in the anterior neural tube starting at embryonic day (E)8.5 permits survival until birth, and at late gestation, these embryos show an absence of the anterior commissure, a failure of the corpus callosum and hippocampal commissure to cross the midline, and defects in corticothalamic and thalamocortical projections [4]. Loss of *Rac1* also leads to a reduction in the proliferation and survival of neural progenitors and a severe defect in tangential migration of inhibitory interneurons [5, 6]. Selective deletion of *Rac1* in the cerebral cortex blocks midline crossing of axons in the corpus callosum and anterior commissure, but there is little or no effect on the development of the corticospinal, corticothalamic, and thalamocortical tracts [7].

The tissue polarity/planar cell polarity (PCP) signaling system is one of several cell-cell signaling systems that control axon guidance. PCP signaling is conserved from insects to mammals, and it has been most intensively studied in the context of epithelia, where it establishes and maintains polarity within the plane of the epithelium relative to global anatomic structures. PCP signaling appears to converge on the cytoskeleton, and a variety of experiments in the context of convergent extension and inner ear sensory hair cell development suggest the direct or indirect involvement of small GTPases as effectors of PCP signaling [8–12].

Mutations in either of two core PCP genes, *Frizzled* (*Fz*)*3* and *Celsr3*, cause profound and virtually identical defects in axon guidance in mice, including defects in corticothalamic, thalamocortical, nigrostriatal, and spinal sensory axon tracts [13–19]. We recently conducted a comprehensive analysis of axonal development in both the central and peripheral nervous systems in *Fz3*^*−/−*^ mice [17, 18], and we were intrigued by the potential similarities between the *Fz3*^*−/−*^ axon guidance defects and the axon guidance defects reported by Chen et al. [4] and Kassai et al. [7] in forebrain-specific *Rac1* knockout mice. Here we present a comprehensive analysis of axonal development in conditional mutants of *Rac1*, including the first analysis of the *Rac1* mutant phenotype in sensory and motor neurons. We have used the same methodologies that were used in our earlier studies of *Fz3* mutants, and the resulting comparisons of *Fz3* and *Rac1* loss-of-function phenotypes reveal several striking similarities.

## Results

### Defects in forebrain axon tracts in *Foxg1-Cre;Rac1*^*CKO*^^*/−*^ embryos

**Fig. 1:**
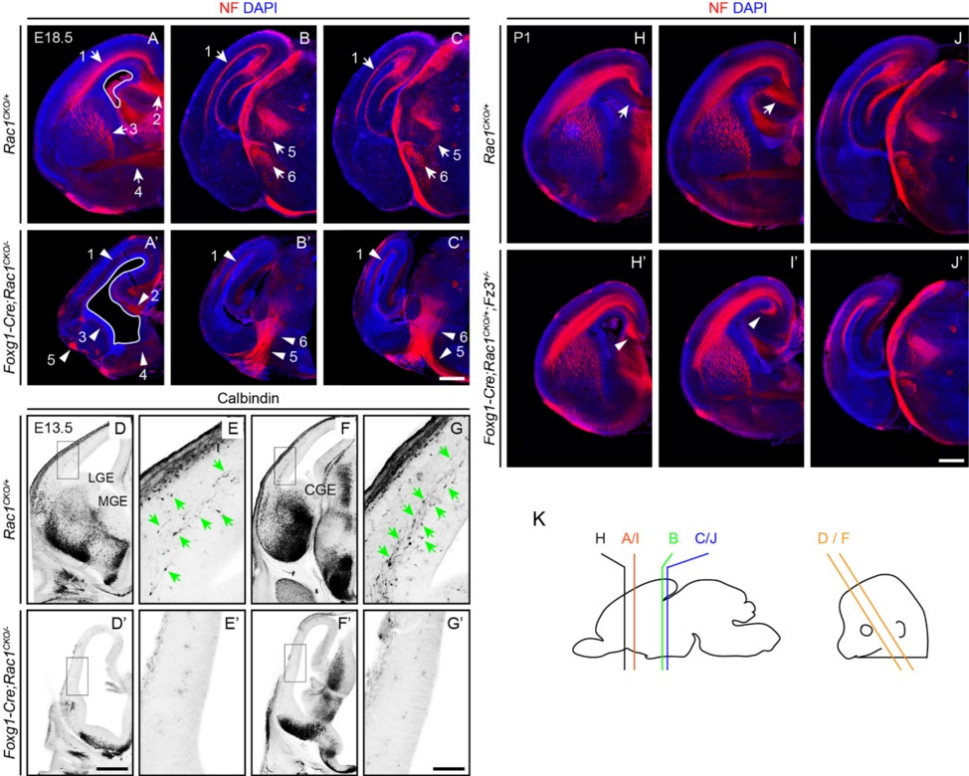
Defects in development of major axon tracts and migration of interneurons in the *Foxg1-Cre;Rac1* ^*CKO/−*^ forebrain. **a**–**c’** Neurofilament (NF) immunostaining of coronal brain sections at E18.5 shows an enlarged lateral ventricle (encircled by a *white line*), absence of multiple axon tracts, and misrouting of thalamocortical axons in the embryonic *Foxg1-Cre;Rac1* ^*CKO/−*^ brain. In the *Foxg1-Cre;Rac1* ^*CKO/−*^ brain, corticospinal axons are partially obscured by misrouted thalamocortical axons. *Numbered white arrows* (control) and *arrowheads* (mutant) point to corresponding axon tracts: *1*, axons in the cortical intermediate zone; *2*, corpus callosum; *3*, axons in the striatum; *4*, anterior commissure; *5*, thalamocortical axons; and *6*, corticospinal tract. *N* = 2 for each genotype. In this and all other figures, images are shown from a representative embryo of the indicated genotype. Scale bar, 500 μm. **d**–**g’** Calbindin immunostaining of E13.5 sections shows the absence of calbindin^+^ interneurons in the *Foxg1-Cre;Rac1* ^*CKO/−*^ neocortex. **e**, **e’**, **g**, **g’** are enlarged views of boxed regions in (**d**, **d’**, **f**, **f’**), respectively. In the WT neocortex, interneurons (*green arrows*) exhibit elongated bipolar morphology parallel to the plane of the cortex, indicative of tangential migration. In the *Foxg1-Cre;Rac1* ^*CKO/−*^ forebrain, calbindin^+^ interneurons are seen in the ventral telencephalon but are not found in the neocortex. The lateral ventricle is already enlarged by this stage. Faint background immunostaining is present in the vasculature. *CGE* caudal ganglionic eminence, *LGE* lateral ganglionic eminence, *MGE* medial ganglionic eminence. *N* = 3 for each genotype. Scale bars: **d’**, 500 μm; **g’**, 100 μm. **h**–**j’** Neurofilament (NF) immunostaining of coronal *Rac1* ^*CKO/+*^ vs. *Foxg1-Cre;Rac1* ^*CKO/+*^ *;Fz3* ^*+/−*^ brain sections at E18.5. The corpus callosum is indicated by a *white arrow* in (**h**, **i**). The corresponding non-crossing fibers are indicated by a *white arrowhead* in (**h’**, **i’**). *N* = 2 for each genotype. Scale bar, 500 μm. **k** Planes of sections in (**a**–**g’**)

**Fig. 2:**
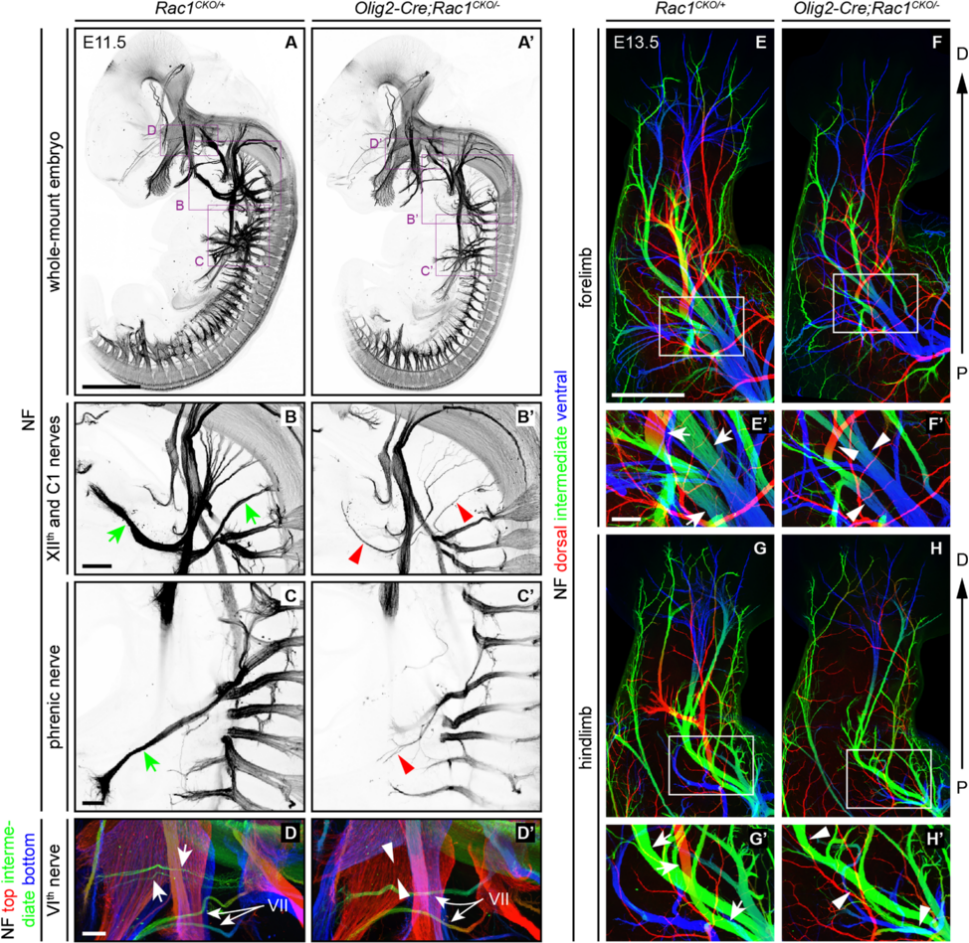
Defects in peripheral nerves in *Olig2-Cre;Rac1* ^*CKO/−*^ embryos. (**a**–**d’**) Neurofilament (NF) immunostaining of whole-mount E11.5 embryos reveals severely thinned XIIth and C1 nerves (*green arrows* in (**b**) vs. *red arrowheads* in (**b’**)) and phrenic nerve (*green arrow* in (**c**) vs. *red arrowhead* in (**c’**)) and a complete absence of the VIth nerve (*arrows* in (**d**) vs. *arrowheads* in (**d’**)) in the *Olig2-Cre;Rac1* ^*CKO/−*^ embryo. The VIIth nerve is unaffected (*lower arrows* in (**d**) and (**d’**)). **b**–**c’** are maximum intensity projections of subsets of complete *Z* stacks. In (**d**) and (**d’**) axons are color coded to represent depth within the *Z* stack, with *red*, *green*, and *blue* representing axons on the near, center, and far sides of the stack, respectively. *N* = 3 WT and *N* = 8 mutants. Scale bars: **a**, 1 mm; **b**, 200 μm; **c** and **d**, 100 μm. **e**–**h’** Neurofilament (NF) immunostaining of whole-mount E13.5 limbs shows thinned dorsal and ventral nerves in *Olig2-Cre;Rac1* ^*CKO/−*^ embryos compared to control forelimbs and hindlimbs. **e’**–**h’** show magnified views of the *boxed regions* in (**e**–**h**). The *red* to *blue* color code represents depth within the *Z* stack, oriented along the dorsal to ventral axis. *Arrows* (control) and *arrowheads* (mutant) indicate corresponding nerves that are thinner in the mutant. *D* distal; *P* proximal. *N* = 14 for each genotype (summing E12.5 and E13.5). Scale bars: **e**, 500 μm; **e’**, 100 μm

To compare axon growth and guidance phenotypes produced by loss of *Rac1* with our earlier studies of *Fz3* loss-of-function mutations in the forebrain, we immunostained coronal sections of E18.5 *Foxg1-Cre;Rac1*^*CKO/−*^ and control *Rac1*^*CKO/+*^ brains for neurofilament and compared them to an analogous set of coronal sections from *Foxg1-Cre;Fz3*^*CKO/−*^ embryos, shown in Figures 1 and 2 of Hua et al. [18]. (*Foxg1-Cre* is expressed prior to E10.5 in the anterior neural tube, leading to Cre-mediated recombination in all or nearly all CNS cell types in the developing telencephalon). Figure 1a–c’ shows that *Foxg1-Cre;Rac1*^*CKO/−*^ forebrains are smaller than littermate controls and they exhibit axon growth and guidance defects that closely resemble the defects seen in *Foxg1-Cre;Fz3*^*CKO/−*^ forebrains (compare to Figure 2B-B” of Hua et al. [18]). In particular, Fig. 1a’–c’ shows that in *Foxg1-Cre;Rac1*^*CKO/−*^ forebrains the lateral ventricles are enlarged, axons are missing from the subcortical zone, the corpus callosum is misrouted away from the midline, axons are missing from the striatum, and the anterior commissure is missing. Among thalamocortical axons, none traverses the striatum to innervate the cortex. Instead, a majority of these axons are misrouted so that they project to the contralateral thalamus, resulting in a dense U-shaped axon tract along the inferior border of the thalamus. The remaining minority of thalamocortical axons are misrouted to the ventral ipsilateral cortex. The misrouting of thalamocortical axons in *Foxg1-Cre;Rac1*^*CKO/−*^ brains is indistinguishable from the phenotype observed in *Foxg1-Cre;Fz3*^*CKO/−*^ brains (compare Fig. 1c’ with Figures 1 and 2 in Hua et al. [18]).

Inhibitory interneurons in the cerebral cortex are born in the medial ganglionic eminence and migrate tangentially across the cortex between E12 and E16 [20]. Using Lhx6.1 and GAD67 as markers at E16.5 and E18.5, Chen et al. [4] observed a nearly complete absence of interneurons in the cerebral cortex in *Foxg1-Cre;Rac1*^*CKO/CKO*^ embryos. Using calbindin as a marker for a subset of interneurons, Fig. 1d–g’ confirms those results by showing that, at E13.5, migrating interneurons are present in the control cortex but are absent from the *Foxg1-Cre;Rac1*^*CKO/−*^ cortex.

To test the possibility that combined haplo-insufficiency for *Fz3* and *Rac1* might reveal a genetic interaction, we compared *Rac1*^*CKO/+*^ vs. *Foxg1-Cre;Rac1*^*CKO/+*^*;Fz3*^*+/−*^ coronal sections of E18.5 brains immunostained for neurofilament. Heterozygous deletion of either *Fz3* or *Rac1* has no detectable effect on brain size or the pattern of neurofilament staining ([13, 14]; Fig. 1a–c, h–j). However, heterozygous deletion of both *Fz3* and *Rac1* in the forebrain (*Foxg1-Cre;Rac1*^*CKO/+*^*;Fz3*^*+/−*^) results in an absence of the corpus callosum and a brain that is reduced to a size intermediate between wild type (WT) controls and *Foxg1-Cre;Rac1*^*CKO/−*^ (Fig. 1h–j’; compare to Fig. 1a–c’). In the *Foxg1-Cre;Rac1*^*CKO/+*^*;Fz3*^*+/−*^ brain, the axons that would normally comprise the corpus callosum collect adjacent to the midline, a defect that is seen in a variety of axon guidance mutants [21]. This genetic interaction is suggestive of a mechanistic link between *Rac1* and *Fz3* function, although it is also possible that it represents an additive effect in a system that is sensitive to genetic perturbation.

### Survey of peripheral nerve defects in *Olig2-Cre;Rac1*^*CKO/−*^ embryos

To broadly survey peripheral nerve phenotypes caused by loss of *Rac1*, we visualized axons in whole-mount E11.5 *Olig2-Cre;Rac1*^*CKO/−*^ embryos by immunostaining for neurofilament (Fig. 2a–d’). *Olig2-Cre* is expressed in many cranial and spinal motor neurons beginning at E9.5 [22–25]. This analysis revealed a severe thinning of the VIth, XIIth, C1 segmental, and phrenic nerves in *Olig2-Cre;Rac1*^*CKO/−*^ embryos, a set of nerves that partially overlaps the set that are thinned in *Fz3*^*−/−*^ and *Olig2-Cre;Fz3*^*CKO/−*^ embryos (compare Fig. 2a–c’ to Figures 1 and 6 in Hua et al. [17]). Whole-mount neurofilament staining of *Olig2-Cre;Rac1*^*CKO/−*^ fore- and hindlimbs at E13.5 shows a severe reduction in the diameter of the dorsal nerve and a more modest reduction in the diameter of the ventral nerve in the hindlimb, with only a modest reduction in the diameter of both dorsal and ventral nerves in the forelimb (Fig. 2e–h’). Among these four nerves, the dorsal nerve in the hindlimb also showed the greatest thinning in *Fz3*^*−/−*^ and *Olig2-Cre;Fz3*^*CKO/−*^ embryos [17].

**Fig. 3:**
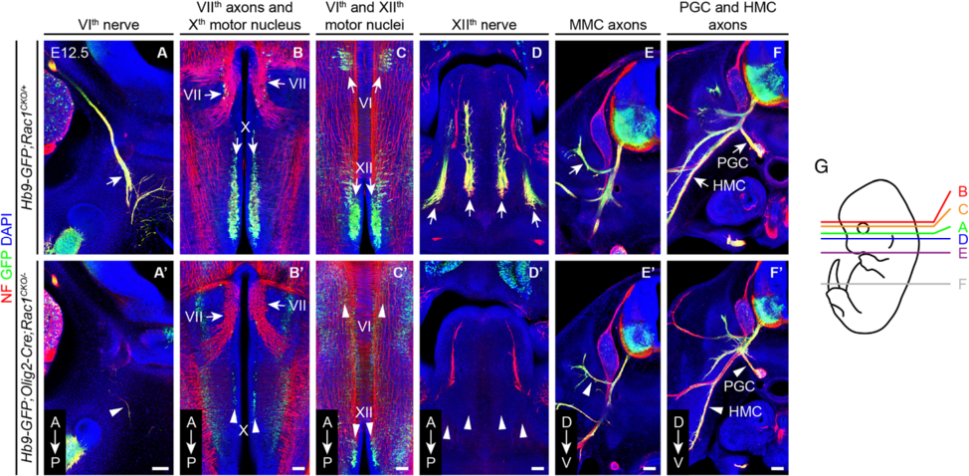
Motor nerve defects in *Olig2-Cre;Rac1* ^*CKO/−*^ embryos revealed by *Hb9-GFP.* **a**–**f’** GFP and neurofilament (NF) immunostaining of E13.5 embryo sections shows a greatly reduced VIth nerve (*arrow* in (**a**) vs. *arrowhead* in (**a’**)); unaffected VIIth nerve axons (*top two arrows* in (**b**) and (**b’**)), decreased numbers of VIth, Xth, and XIIth motor neurons (*lower two arrows* in (**b**) and *arrows* in (**c**) vs. *arrowheads* in (**b’**) and (**c’**)); failure of the XIIth nerve to innerve the tongue (*arrows* in (**d**) vs. *arrowheads* in (**d’**)); and thinned MMC (*arrow* in (**e**) vs. *arrowhead* in (**e’**)), PGC (*upper arrow* in (**f**) vs. *upper arrowhead* in (**f’**)), and HMC (*lower arrow* in (**f**) vs. *lower arrowhead* in (**f’**)) axon bundles. *HMC* hypaxial motor column, *MMC* medial motor column, *PGC* preganglionic motor column. *A* anterior, *P* posterior, *D* dorsal, *V* ventral. *N* = 2 for each genotype. Scale bars, 100 μm. **g** Planes of sections in (**a**–**f’**)

To distinguish motor vs. sensory axon contributions to the peripheral nerve defects, the *Hb9-GFP* reporter was used to selectively visualize cranial and spinal motor axons in *Olig2-Cre;Rac1*^*CKO/−*^ and control *Rac1*^*CKO/+*^ embryos (Fig. 3). This analysis revealed a complete or nearly complete loss of the motor component of the VIth and XIIth nerves (Fig. 3a vs. 3a’ and Fig. 3d vs. 3d’) and a massive reduction in the number of motor neurons in the VIth, Xth, and XIIth cranial nerve nuclei at E12.5 in *Hb9-GFP;Olig2-Cre;Rac1*^*CKO/−*^ embryos (Fig. 3b vs. 3b’ and Fig. 3c vs. 3c’). We did not assess motor axons within the Xth cranial nerve, but we presume that their numbers are correspondingly reduced. Axons within the VIIth nerve were *Hb9-GFP* negative and were unaffected in *Olig2-Cre;Rac1*^*CKO/−*^ embryos (Fig. 3b vs. 3b’). In the E12.5 *Hb9-GFP;Olig2-Cre;Rac1*^*CKO/−*^ spinal cord, motor axons originating in the medial motor column (MMC), hypaxial motor column (HMC), and preganglionic motor column (PGC) were thinned relative to their *Hb9-GFP;Rac1*^*CKO/+*^ counterparts (Fig. 3e vs 3e’ and Fig. 3f vs. 3f’). These data establish an essential role for *Rac1* in the development and survival of a large number of cranial and spinal motor neurons.

### Quantitative analysis of limb motor axon defects and spinal motor neuron loss in *Olig2-Cre;Rac1*^*CKO/−*^ embryos

**Fig. 4:**
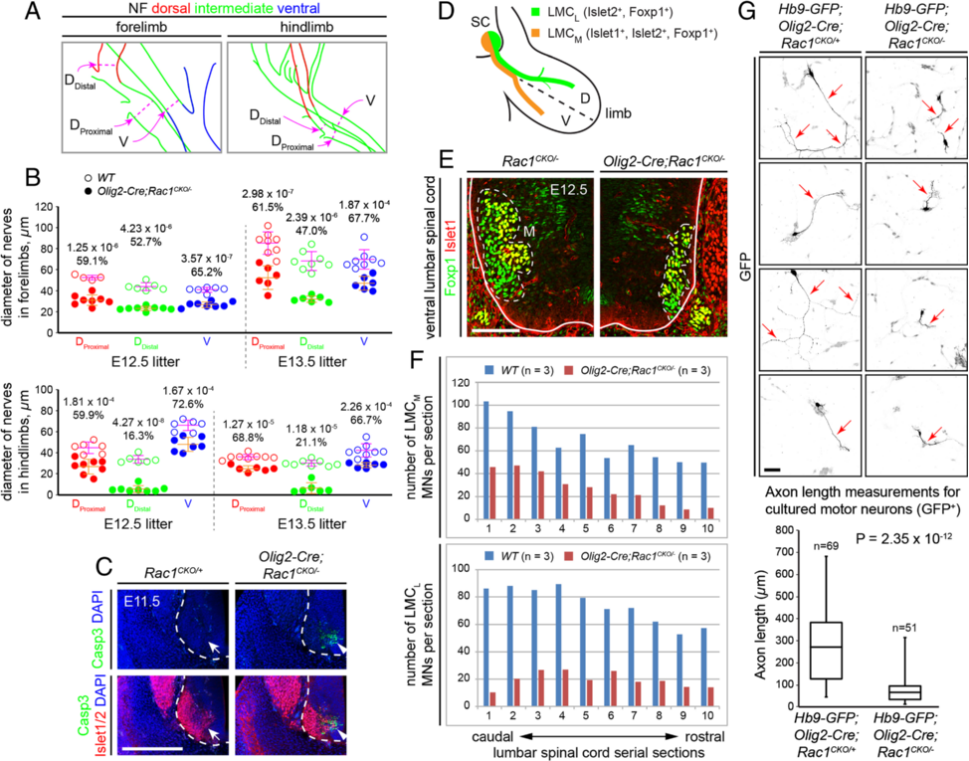
Reduced diameters of limb nerves and reduction in the number of LMC motor neurons in *Olig2-Cre;Rac1* ^*CKO/−*^ embryos. **a** Diagrams traced from Fig. 2e’, g’ showing where the nerve diameters were measured. *D*_Proximal_ and *D*_Distal_, measurements of dorsal nerve diameter immediately proximal and distal, respectively, to the point where *Fz3* ^*−/−*^ dorsal axons stall. *V*, measurement of ventral nerve diameter at a similar distance from the plexus. **b** At the locations shown in (**a**), the nerve diameters were measured from neurofilament-immunostained whole-mount forelimbs (*top*) and hindlimbs (*bottom*) from one litter at E12.5 and a second litter at E13.5 (*left* and *right*, respectively, separated by *gray dashed lines*). Numbers above each set of data points are as follows: (*top*) *P* values for the WT littermate vs. *Olig2-Cre;Rac1* ^*CKO/−*^ comparison (Student’s two-tailed two-sample unequal variance *t*-tests) and (*bottom*) the average thickness of *Olig2-Cre;Rac1* ^*CKO/−*^ nerves as a percentage of the WT controls. **c** Motor neuron apoptosis visualized with cleaved caspase3 and Islet1/2 immunostaining in cross sections of E11.5 lumbar spinal cords. The ventrolateral region of the spinal cord is shown with the left border of the spinal cord demarcated by a *white dashed line*. Very few apoptotic cells are present in *Rac1* ^*CKO/+*^ controls (*left*), whereas cleaved caspase3 is seen in multiple Islet1/2^+^ cells in the *Olig2-Cre;Rac1* ^*CKO/−*^ ventral spinal cord (*right*). *N* = 3 for each genotype. Scale bar, 500 μm. **d** Diagram showing the expression pattern of transcription factors in LMC_L_ and LMC_M_ motor neurons. *SC* spinal cord, *D* dorsal, *V* ventral. Adopted from Figure 5A of Hua et al. [17]. **e** LMC_M_ and LMC_L_ motor neurons in the ventrolateral spinal cord were identified as Islet1^+^/Foxp1^+^ and Islet1^−^/Foxp1^+^ populations, respectively, in cross sections of E12.5 *Rac1* ^*CKO/−*^ (*left panel*; lateral is to the left) and *Olig2-Cre;Rac1* ^*CKO/−*^ (*right panel*; lateral is to the right) lumbar spinal cords. *White dashed lines* encircle the LMC. The *solid white line* demarcates the border of the spinal cord. *L* lateral, *M* medial. Scale bar, 100 μm. **f** Quantification of the number of LMC_M_ (*top*) and LMC_L_ (*bottom*) motor neurons per 14-μm-thick cross section of E12.5 lumbar spinal cords. Motor neurons were counted from 10 serial sections and averaged from three pairs of WT (*blue*) and *Olig2-Cre;Rac1* ^*CKO/−*^ (*red*) littermates, with adjacent counted sections separated by four uncounted sections. **g** *Top*, GFP immunostaining shows defects in axon growth among primary spinal cord motor neurons in dissociated cell cultures from E11.5 *Hb9-GFP;Olig2-Cre;Rac1* ^*CKO/−*^ (*right*) and littermate control *Hb9-GFP;Olig2-Cre;Rac1* ^*CKO/+*^ (*left*) embryos. *Red arrows*, axons and axon branches. *Bottom*, quantification of axon lengths. Scale bar, 100 μm

In earlier work, we quantified a distinctive axon stalling phenotype at the base of the limbs in *Fz3*^*−/−*^ embryos by measuring dorsal and ventral nerve diameters in whole mounts of neurofilament-stained limbs at E12.5 and E13.5 [17]. For the dorsal nerves, measurements were made at locations proximal and distal to the point of axon stalling (see Figure 4 in Hua et al. [17]). We have performed the same analysis on E12.5 and E13.5 *Olig2-Cre;Rac1*^*CKO/−*^ and WT littermates (Fig. 4a, b). At both ages, *Olig2-Cre;Rac1*^*CKO/−*^ fore- and hindlimb ventral nerve diameters were reduced, on average, to 65–73 % of the diameters of littermate controls. Similarly, the *Olig2-Cre;Rac1*^*CKO/−*^ fore- and hindlimb dorsal nerve diameters proximal to the *Fz3*^*−/−*^ stalling point were reduced, on average, to 59–69 % of the diameters of littermate controls. Interestingly, the *Olig2-Cre;Rac1*^*CKO/−*^ dorsal nerve diameters distal to the *Fz3*^*−/−*^ stalling point were reduced, on average, in the forelimb to 47–53 % of the control diameters and in the hindlimb to 16–21 % of the control diameters. These data indicate that in *Olig2-Cre;Rac1*^*CKO/−*^ embryos there is a severe axon stalling defect in the hindlimb dorsal nerve and a moderate axon growth defect in the three other limb nerves that were quantified in Fig. 4a, b. These patterns of nerve diameter reduction closely match the patterns observed in *Fz3*^*−/−*^ limbs, both qualitatively and quantitatively.

Our earlier analysis of *Fz3*^*−/−*^ embryos showed that axon stalling leads to a secondary loss of spinal and cranial motor neurons. In the late E11.5 lumbar spinal cord, motor neuron loss was largely limited to the lateral division of the lateral motor column (LMC; Figure 5 in Hua et al. [17]). A similar analysis of *Olig2-Cre;Rac1*^*CKO/−*^ lumbar spinal cords showed an increase in the number of apoptotic motor neurons at E11.5, as determined by immunostaining for cleaved caspase3 (Fig. 4c), and a reduction in motor neurons in both the medial and lateral divisions of the lateral motor column (LMC_M_ and LMC_L_) relative to littermate controls (Fig. 4d–f). Cell loss in both divisions rather than a single division implies the presence of developmental defects in the *Olig2-Cre;Rac1*^*CKO/−*^ spinal cord that are broader than the defects in the *Fz3*^*−/−*^ spinal cord.

To assess the autonomy of the motor axon growth defects in *Olig2-Cre;Rac1*^*CKO/−*^ embryos, dissociated cells were cultured from E11.5 spinal cords of *Hb9-GFP;Olig2-Cre;Rac1*^*CKO/−*^ and littermate control *Hb9-GFP;Olig2-Cre;Rac1*^*CKO/+*^ embryos, and among GFP^+^ neurons, axon lengths were quantified after 2 days in culture. In a sample of 51 *Hb9-GFP;Olig2-Cre;Rac1*^*CKO/−*^ and 69 control axons, the axons of *Rac1*-deficient motor neurons had a mean length approximately one third of the length of the control group (*P* < 10^−11^) (Fig. 4g). Thus, loss of *Rac1* impairs axon growth in a cell-autonomous manner. In dissociated neuronal cultures, a similar cell-autonomous axon growth defect was observed among *Rac1*-deficient cerebellar granule cells [26], but no axon growth defect was observed among *Rac1*-deficient cerebral cortical pyramidal neurons [4]. The results of Chen et al. [4] could reflect the presence of compensating Rho family members in cortical neurons.

### A requirement for *Rac1* in sensory neurons: defects in dorsal root ganglia and in cranial nerves and ganglia in *Wnt1-Cre;Rac1*^*CKO/−*^ embryos

**Fig. 5:**
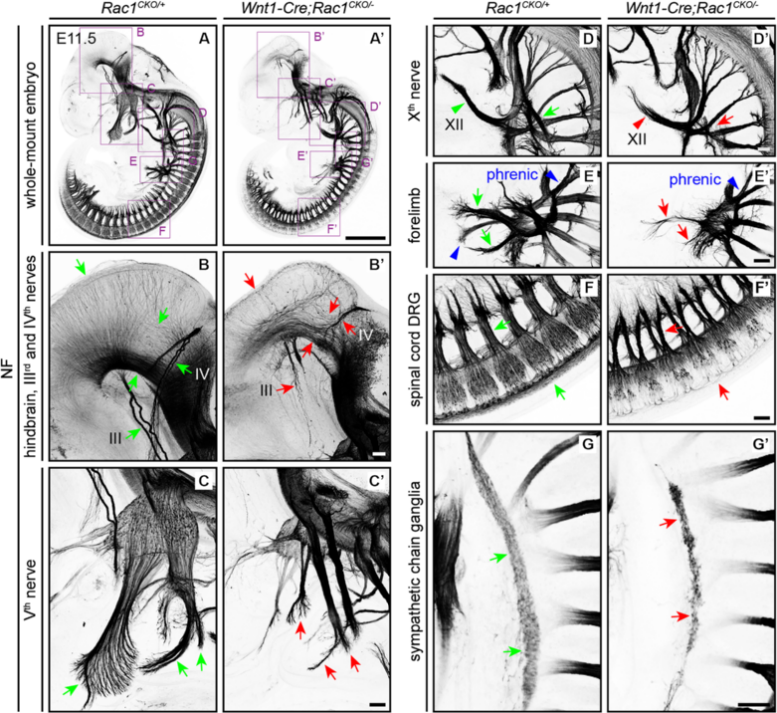
Axon tract and peripheral nerve defects in *Wnt1-Cre;Rac1* ^*CKO/−*^ embryos. **a**–**g’** Neurofilament (NF) immunostaining of whole-mount E11.5 embryos shows that in *Wnt1-Cre;Rac1* ^*CKO/−*^ embryos, multiple axon tracts are aberrant including the following: diffuse axonal projections in the hindbrain and the IIIrd and IVth nerves (*green arrows* in (**b**) vs. *red arrows* in (**b’**)), the Vth nerve (*green arrows* in (**c**) vs. *red arrows* in (**c’**)), the Xth (*green arrow* in (**d**) vs. *red arrow* in (**d’**)) and XIIth nerves (*green arrowhead* in (**d**) vs. *red arrowhead* in (**d’**)), multiple nerves in the forelimb (*green arrows* in (**e**) vs. *red arrows* in (**e’**)), and ventral axons from the DRG (*green arrows* in (**f**) vs. *red arrows* in (**f’**)). The sympathetic chain ganglia are reduced in size (*green arrows* in (**g**) vs. *red arrows* in (**g’**)). To show the individual hindbrain axons, the neurofilament signal intensity was increased in the (**b**) and (**b’**) images. **d**–**e’**, **g**, **g’** are maximum intensity projections of a subset of *Z* stacks containing the entire nerves or chain ganglia. *N* = 3 for each genotype. Scale bars: **a’**, 1 mm; **b’**–**g’**, 100 μm

To explore the role of Rac1 in sensory neurons, which are not sites of Cre-mediated recombination by the *Olig2-Cre* driver, we examined the phenotype of E11.5 *Wnt1-Cre;Rac1*^*CKO/−*^ embryos. By whole-mount neurofilament immunostaining at E11.5, *Wnt1-Cre;Rac1*^*CKO/−*^ embryos exhibited severe diminutions in the IIIrd, IVth, and Vth cranial nerves and a distal stalling of the XIIth cranial nerve (Fig. 5a–d’). Additionally, E11.5 *Wnt1-Cre;Rac1*^*CKO/−*^ embryos showed a reduction in the number of axons in the nerves originating in the dorsal root ganglion (DRG) and the sympathetic chain ganglia (Fig. 5e–g’). In *Olig2-Cre;Rac1*^*CKO/−*^ embryos, the IIIrd, IVth, and Vth cranial nerves were unaffected (Fig. 2a, a’).

**Fig. 6:**
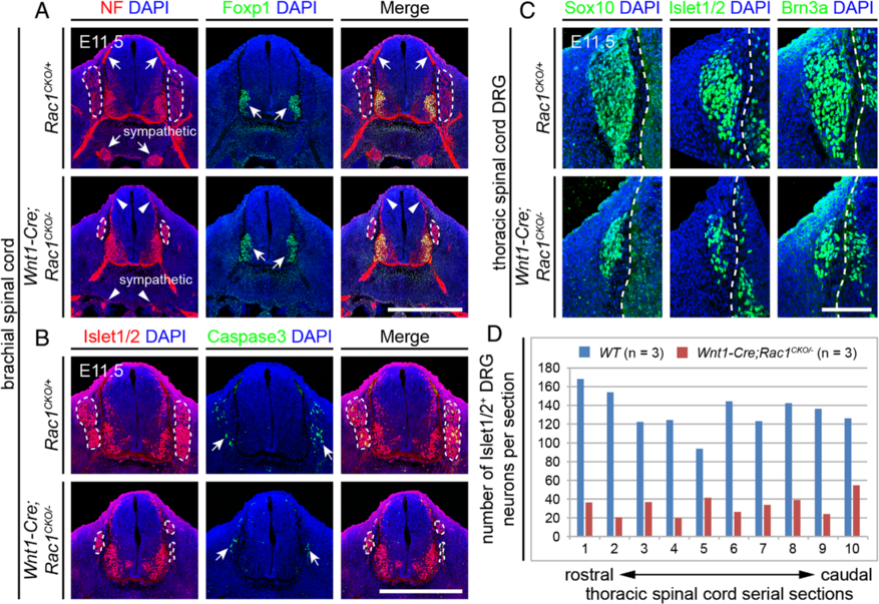
Abundance of DRG neurons in *Wnt1-Cre;Rac1* ^*CKO/−*^ embryos. **a** Neurofilament (NF) and Foxp1 immunostaining of E11.5 brachial spinal cord and DRG cross sections. In the *Wnt1-Cre;Rac1* ^*CKO/−*^ embryo, the DRG (encircled by *white dashed lines*) are reduced in size, the axons projecting from the DRG to the dorsal spinal cord (upper pair of *arrows* vs. *arrowheads*) are reduced in diameter, and the sympathetic ganglia are reduced in size (lower pair of *arrows* vs. *arrowheads* in the *left panels*). At this age, LMC motor neurons are unaffected in *Wnt1-Cre;Rac1* ^*CKO/−*^ embryos (*arrows* in *middle panels*). *N* = 2 for each genotype. Scale bar, 500 μm. **b** Islet1/2 and cleaved caspase3 immunostaining of E11.5 brachial spinal cord and DRG cross sections showing a similar density of apoptotic cells in *Rac1* ^*CKO/+*^ and *Wnt1-Cre;Rac1* ^*CKO/−*^ DRG, which are encircled by *white dashed lines* and indicated by *paired arrowheads* in the *middle panels. N* = 2 for each genotype. Scale bar, 500 μm. **c** Loss of *Rac1* does not affect differentiation of neurons in the DRG (in the center of each image), as determined by Sox10, Islet1/2, and Brn3a immunostaining of cross sections from E11.5 *Wnt1-Cre;Rac1* ^*CKO/−*^ thoracic DRG. The lateral edge of the spinal cord, which is on the right side of each image, is delineated by *dashed white lines. N* = 1 for each genotype. Scale bar, 100 μm. **d** Quantification of Islet1/2^+^ DRG neurons per 14-μm-thick cross section of E11.5 thoracic DRG. DRG sensory neurons were counted from 10 serial sections and averaged from three pairs of WT control (*blue*) and *Wnt1-Cre;Rac1* ^*CKO/−*^ (*red*) littermates, with adjacent counted sections separated by four uncounted sections

*Wnt1-Cre* is expressed in migrating neural crest cells, including those that form the DRG and cranial sensory ganglia [27]. As Rac1 acts in both cell migration and axon guidance, it would not be surprising to find that defects in one or both of these processes affect sensory neuron development. To assess Rac1 effects at the earliest stages of DRG development, we compared the DRG in coronal sections of *Wnt1-Cre;Rac1*^*CKO/−*^ and littermate control embryos at E11.5. This comparison revealed a dramatic reduction in the size of the DRG and number of DRG neurons in the brachial and thoracic regions in *Wnt1-Cre;Rac1*^*CKO/−*^ embryos, but there was no difference between mutant and control embryos in the expression of DRG markers (Brn3a, Islet1/2, Sox10), and there was little or no difference between mutant and control embryos in the relative density of DRG neurons with activated caspase3 at this stage (Fig. 6a–d).

In earlier work, we found that defective migration of DRG progenitors in *Fz3*^*−/−*^ embryos produces a characteristic accumulation of neurons expressing DRG markers (neurofilament, Brn3a, and Islet1/2, or Sox10) in the dorsal neural tube at E11.5 and E12.5 [17]. These cell clusters were not observed in *Wnt1-Cre;Rac1*^*CKO/−*^ embryos (compare Fig. 5a, a’, f, f’ with Figure 3 of Hua et al. [17]). At present, it is not clear whether the primary defect in *Wnt1-Cre;Rac1*^*CKO/−*^ DRG development is in neural crest migration, DRG axon growth and/or guidance, or some combination of the two, since we have not identified the missing neural crest cells. However, it is apparent that DRG neurons are affected early in their development, since they are already missing from the DRG at E11.5.

### Defects in retinal ganglion cell axon guidance and survival in *Pax6-alpha-Cre;Rac1*^*CKO/−*^ and *Six3-Cre;Rac1*^*CKO/−*^ mice

**Fig. 7:**
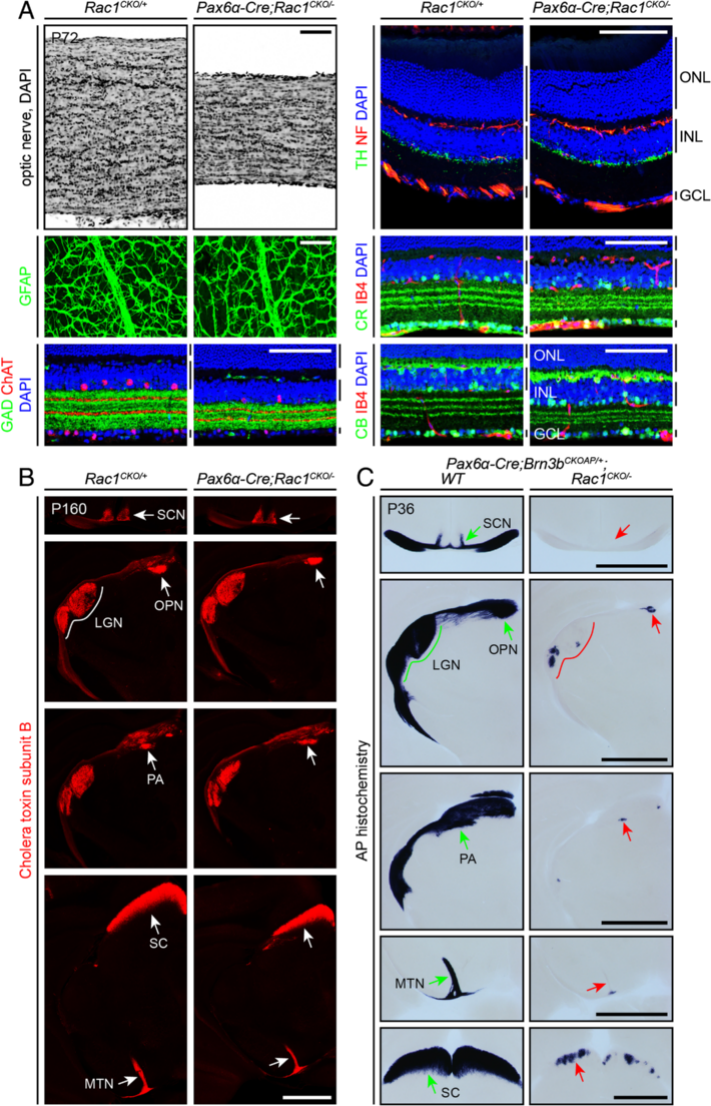
Partial loss of *Rac1* in the retina leads to a cell-autonomous loss of retinal ganglion cell projections to retino-recipient targets in the brain. **a** *Rac1* inactivation in the peripheral retina by *Pax6*α*-Cre* results in an optic nerve that is reduced approximately twofold in diameter and an inner plexiform layer (IPL) that is modestly reduced in thickness. *Pax6*α*-Cre;Rac1* ^*CKO/−*^ retinas show the normal two tiers of intraretinal capillaries (IB4 staining), and GABAergic (GAD), cholinergic (ChAT), dopaminergic [i.e., tyrosine hydroxylase (TH)]-expressing, calretinin^+^ (CR), and calbindin^+^ (CB) amacrine cells and calbindin^+^ horizontal cells stratify within the correct retinal laminae. Glial fibrillary acidic protein (GFAP) immunostaining to visualize astrocytes is shown as a retina flat mount; all other retina images are transverse sections. The TH, neurofilament (NF), and DAPI images are from the central retina, where *Cre* expression is minimal. *ONL* outer nuclear layer, *INL* inner nuclear layer, *GCL* ganglion cell layer. Bars to the right of the cross-section images: *upper bar* (ONL), *middle bar* (INL), *lower bar* (GCL). *N* = 3 for each genotype. Scale bars, 100 μm. **b** Central projections of RGC axons in adult *Pax6*α*-Cre;Rac1* ^*CKO/−*^ mice target the major retino-recipient areas as revealed by anterograde labeling by intraocular injection of cholera toxin subunit B. Major retino-recipient nuclei are indicated by *arrows* or a *line* for the LGN. *LGN* lateral geniculate nucleus, *MTN* medial terminal nucleus, *OPN* olivary pretectal nucleus, *PA* pretectal area, *SC* superior colliculus, *SCN* suprachiasmatic nucleus. *N* = 2 for each genotype. Scale bar, 1 mm. **c** Alkaline phosphatase (AP) histochemistry reveals the central projections of *Cre*-expressing RGC axons in adult *Pax6*α*-Cre;Brn3b* ^*CKOAP/+*^ and *Pax6*α*-Cre;Rac1* ^*CKO/−*^ *;Brn3b* ^*CKOAP/+*^ mice. Major retino-recipient nuclei are indicated by *green* and *red arrows* or a *line* for the LGN and labeled as in panel (**b**). *N* = 2 for each genotype. Scale bars, 1 mm

The growth of retinal ganglion cell (RGC) axons to their central targets presents a favorable system in which to study long-range axon guidance [28]. Eliminating Rac1 predominantly in the peripheral retina beginning at ~E9.5 with the *Pax6-alpha-Cre* driver [29] does not affect the viability or overall health of the embryo or postnatal mouse and thus permits a phenotypic analysis in adulthood. Figure 7a shows that, in adult *Pax6-alpha-Cre;Rac1*^*CKO/−*^ mice, the diameter of the optic nerve and the thickness of the inner retina (in the retinal periphery) are both reduced, consistent with the expression of the *Pax6-alpha-Cre* transgene in the periphery but not the central ~50 % of the retina. Within the thinned inner retina, the pattern of lamination and the locations and structures of major cell types—including cholinergic, GABAergic, dopaminergic, calbindin-expressing, and calretinin-expressing amacrine cells, and horizontal cells, blood vessels, and astroctyes—appear essentially normal (Fig. 7a). These data closely resemble the phenotype that results from the selective loss of the majority of RGCs, as observed, for example, in *Brn3b*^*−/−*^, *Math5*^*−/−*^, and *Brn3b*^*−/−*^*;Brn3c*^*−/−*^ mice [30–34].

Intraocular injection of fluorescent cholera toxin subunit B in adult *Pax6-alpha-Cre;Rac1*^*CKO/−*^ mice shows an essentially normal pattern of innervation of RGC axon targets, including the suprachiasmatic nucleus (SCN), lateral geniculate nucleus (LGN), olivary pretectal nucleus (OPN), pretectal area (PA), superior colliculus (SC), and the medial terminal nucleus (MTN) (Fig. 7b). These data are consistent with the expectation (based on the pattern of *Pax6-alpha-Cre* expression) that RGCs within the central ~50 % of the retina should retain *Rac1* function. To selectively visualize the axons of RGCs that had lost Rac1, we introduced a Cre-activated *alkaline phosphatase* (*AP*) knock-in allele that is expressed exclusively in RGCs (*Brn3b*^*CKOAP*^; [35]). In the peripheral retina, *Pax6-alpha-Cre* expression is predicted to lead to Cre-mediated recombination of both *Rac1*^*CKO*^ and *Brn3b*^*CKOAP*^, simultaneously eliminating *Rac1* and activating expression of *AP* in RGCs. As seen in the left column of Fig. 7c, control brains from adult *Pax6-alpha-Cre;Brn3b*^*CKOAP/+*^ mice show AP histochemical staining in the same set of retino-recipient areas that were labeled by intraocular injection with cholera toxin subunit B (Fig. 7b). In contrast, the right column of Fig. 7c shows that brains from adult *Pax6-alpha-Cre;Brn3b*^*CKOAP/+*^*;Rac1*^*CKO/−*^ mice exhibit almost no AP staining in retino-recipient regions. We ascribe the occasional presence of focal zones of AP activity in retino-recipient territories to rare RGCs in which the *AP* reporter was activated by Cre-mediated recombination but the *Rac1*^*CKO*^ allele was not recombined. These data indicate that loss of *Rac1* leads to a severe deficiency in RGC axon growth, guidance, and/or RGC survival. The defect appears to be cell-autonomous since it is not corrected by neighboring *Rac1*^*+/−*^ RGCs that develop normally.

**Fig. 8:**
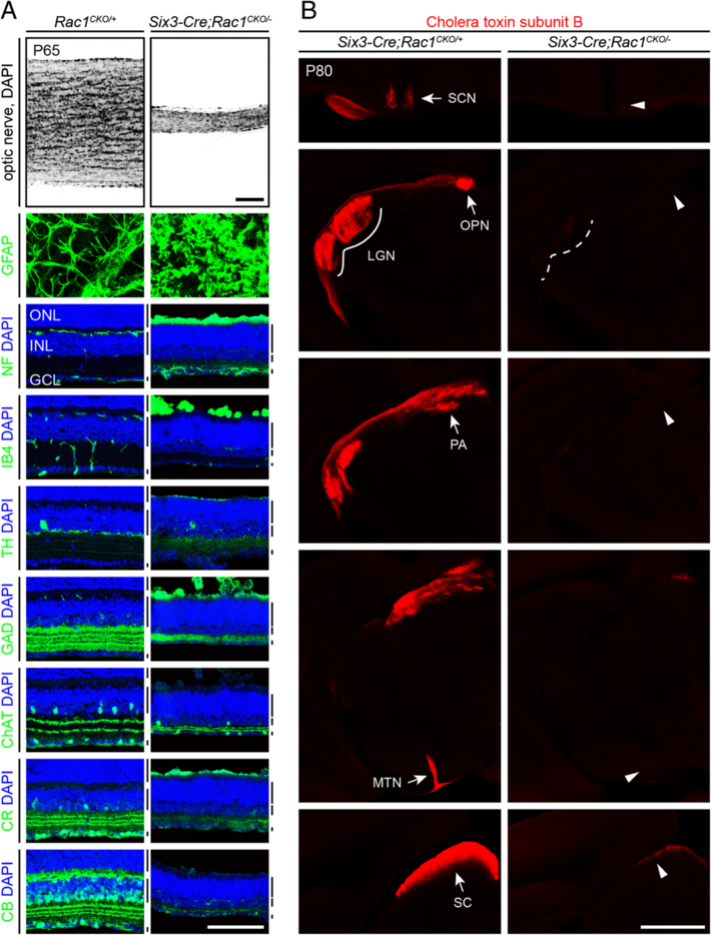
Complete loss of *Rac1* in the retina leads to a severe loss of inner retinal neurons and a nearly complete loss of retinal ganglion cell projections to retino-recipient targets in the brain. **a** *Rac1* inactivation by *Six3-Cre* results in an approximately fivefold reduction in optic nerve diameter, a severe reduction in INL, IPL, and GCL thickness and inner retinal cell number, disorganization of intra-retinal capillaries (IB4), and activation of astrocytes (GFAP). The density of tyrosine hydroxylase (TH)-expressing, GABAergic (GAD), cholinergic (ChAT), calretinin^+^ (CR), and calbindin^+^ (CB) amacrine cell bodies and processes are reduced. GFAP immunostaining is shown as a retina flat mount; all other retina images are transverse sections. *ONL* outer nuclear layer, *INL* inner nuclear layer, *GCL* ganglion cell layer. Bars to the right of the cross-section images: *upper bar* (ONL), *middle bar* (INL), *lower bar* (GCL). *N* = 3 for each genotype. Scale bars, 100 μm. **b** Dramatically reduced innervation of brain targets by RGCs in adult *Six3-Cre;Rac1* ^*CKO/−*^ mice, revealed by anterograde labeling of optic tract axons with intraocular injection of cholera toxin subunit B. *LGN* lateral geniculate nucleus, *MTN* medial terminal nucleus, *OPN* olivary pretectal nucleus, *PA* pretectal area, *SC* superior colliculus, *SCN* suprachiasmatic nucleus. *N* = 2 for each genotype. Scale bar, 1 mm

A more severe retinal phenotype is observed when Cre-mediated deletion of *Rac1* is driven by *Six3-Cre*, a transgene that is expressed in all cells in the neural retina beginning at ~E11 [36]. In adult *Six3-Cre;Rac1*^*CKO/−*^ retinas, the optic nerve is reduced to <20 % of the WT diameter and the inner nuclear layer and inner plexiform layer are dramatically thinned (Fig. 8a). A variety of retinal cell types are present but with substantially distorted morphologies and reduced abundances (Fig. 8a). These include cholinergic, GABAergic, dopaminergic, calbindin-expressing, and calretinin-expressing amacrine cells. Horizontal cells, marked by neurofilament and calbindin expression, appear to be almost completely absent. Neurofilament immunostaining shows accumulation of neurofilament at the inner face of the retina, and glial fibrillary acidic protein (GFAP) immunostaining of retina flat mounts shows enhanced staining in a pattern consistent with reactive astrocytes. By DAPI staining, the numbers of cells in the ganglion cell layer and the inner nuclear layer are reduced to <50 % of the WT level, but the outer nuclear layer is largely unaffected. Tracing adult RGC axons by intraocular injection of fluorescent cholera toxin subunit B in adult *Six3-Cre;Rac1*^*CKO/−*^ retinas shows an almost complete absence of RGC axons in retino-recipient regions (Fig. 8b). At P0, the number of RGCs is similar to the number in WT control retinas, as determined by immunostaining for transcription factor Brn3a (data not shown); earlier work demonstrated that RGC cell death secondary to axon pathfinding defects occurs at ~P10 [37].

Taken together, these data imply a central role for Rac1 in inner retinal development. Based on (1) the cell-autonomous failure of axons from *Rac1*-null RGCs to innervate central targets, (2) the well-established dependence of RGC survival on innervation of central targets (e.g., [37]), and (3) the well-established secondary loss of amacrine cells in mice with primary defects that lead to RGC loss (as seen in *Brn3b*^*−/−*^, *Math5*^*−/−*^, and *Brn3b*^*−/−*^*;Brn3c*^*−/−*^ mice), the simplest interpretation of the *Pax6-alpha-Cre;Rac1*^*CKO/−*^ and *Six3-Cre;Rac1*^*CKO/−*^ retinal phenotypes is that *Rac1* is required in RGCs where it plays an essential role in axon growth. Under this interpretation, the observations in Figs. 7 and 8 imply that loss of *Rac1* in a subset of RGCs (*Pax6-alpha-Cre;Rac1*^*CKO/−*^) leads to axon growth defects and eventual death in this RGC subset and the secondary loss of a relatively small fraction of amacrine cells, whereas loss of *Rac1* in the vast majority of RGCs in *Six3-Cre;Rac1*^*CKO/−*^ retinas leads to axon growth defects and eventual death in nearly all RGCs and the secondary loss of a large fraction of amacrine cells. In *Six3-Cre;Rac1*^*CKO/−*^ retinas, in which *Cre* is expressed in retinal astrocytes, we also observe defects in postnatal retinal vascularization, which involves endothelial cell growth on an astrocyte scaffold, and this could account for part of the axon development and inner retinal phenotypes. However, these considerations do not apply to the *Pax6-alpha-Cre;Rac1*^*CKO/−*^ retina, as *Pax6-alpha-Cre* is not expressed in retinal astrocytes [38].

## Discussion

This study provides a systematic analysis of the consequences of *Rac1* deletion in neurons in the telencephalon, cranial and spinal motor columns, sensory ganglia, and retina. In each of these neuronal populations, we observe defects in axon growth and/or guidance with loss of *Rac1*. Among motor neurons and retinal ganglion cells, these defects are associated with neuronal loss, consistent with the known dependence of neuronal survival on axon targeting. Additionally, we observe cell migration defects among telencephalic interneurons and a large reduction in the number of DRG neurons at E11.5. This early DRG phenotype presumably reflects a defect in the proliferation and/or migration of DRG progenitors from the neural crest. In contrast with earlier studies that used dominant negative or constitutively active mutants of Rac1 or other Rho family GTPases (for example, [39–44]), the loss-of-function approach used here does not have the potential to perturb signaling by closely related small GTPases or their effectors, and therefore, the observed phenotypes can be confidently assigned to Rac1.

### The breadth of Rac1 action in nervous system development

The cell biological processes that underlie nervous system development can be roughly divided into seven general classes: (1) cell proliferation; (2) cell migration; (3) axon growth, guidance, and branching; (4) dendritic growth and branching; (5) synaptogenesis; (6) remodeling/plasticity; and (7) cell survival or death. Rac1 and related Rho GTPases play essential roles in each of these processes, as briefly summarized in points (1–7) below. (1) Rac1 in the embryonic forebrain is required for proliferation of neural progenitors [5, 6], and in the adult, it is required for learning-evoked neurogenesis in the hippocampus [45, 46]. (2) In the telencephalon, Rac1 is required for tangential migration of interneurons from the medial ganglionic eminence ([4, 47]; this work), as are RhoA and Cdc42 [48]. In the rostral migratory stream, Rac1 is required for neuronal migration [49]. (3) Down-regulation of Rac1 activity by the breakpoint cluster region GTPase activating protein decreases dendritic branching in vivo and in vitro [50], and constitutive activation of Rac1 increases dendritic growth and branching in the cortex and hippocampus [51, 52]. (4) In *Drosophila*, Rac1 and the closely related Rho GTPases Rac2 and Mtl control axon growth, guidance, and branching [8, 53], and in the mouse, Rac1 is required widely in the central and peripheral nervous systems for axon growth and guidance ([4, 7]; this work). In the hippocampus, Rac1 is additionally involved in axon pruning [54]. (5) In *C. elegans*, the reorganization of the actin cytoskeleton and the clustering of synaptic vesicles in response to netrin signaling require Rac1 [55], and in mice, EphB signaling via the GEF Tiam requires Rac1 for excitatory spine remodeling [56]. (6) The synaptic functions of Rac1 continue throughout life, as determined by defects in hippocampal plasticity and impaired spatial memory in mice with defects in Rac1 signaling [57, 58]. (7) Finally, Rac1 regulates cell survival by mechanisms that may include both direct and indirect effects. In the ventricular zone and subventricular zone, deletion of Rac1 leads to accelerated cell cycle exit and reduced survival of progenitors, an effect that may reflect Rac1 regulation of cell cycle progression and apoptosis [5, 6]. Among cranial and spinal motor neurons and RGCs, cell death may be secondary to defects in axon growth and guidance (this work).

### Comparison of Rac1 and Fz3/Celsr3 phenotypes

As noted in the “Background” section, the initial impetus for this study was to compare *Rac1* and *Fz3* phenotypes. With these two phenotypes now defined in detail using the same methodologies, it is apparent that they share a number of features ([4, 7, 13, 14, 17, 18], this work). Most prominently, the distinctive misrouting of thalamocortical axons—with the majority projecting to the contralateral thalamus and a minority projecting to the ventral cortex—is virtually identical in *Foxg1-Cre;Rac1*^*CKO/−*^, *Fz3*^*−/−*^, and *Celsr3*^*−/−*^ embryos, as well as in *Foxg1-Cre;Fz3*^*CKO/−*^ and *Foxg1-Cre;Celsr3*^*CKO/CKO*^ embryos [15, 16], suggesting that there may be a stereotyped default pathway for these axons if they do not traverse the normal route through the internal capsule. Mutations in *Rac1*, *Fz3*, and *Celsr3* also lead to a loss of the corticospinal tract, the corticothalamic tract, and the anterior commissure. However, the loss of the corpus callosum in *Foxg1-Cre;Rac1*^*CKO/−*^ embryos is not seen in *Fz3*^*−/−*^ or *Celsr3*^*−/−*^ embryos, where the corpus callosum is only mildly affected. Motor-neuron-specific loss of Rac1 in *Olig2-Cre;Rac1*^*CKO/−*^ embryos produces a distinctive stalling of axons within the dorsal nerve of the hindlimb but a much milder loss of axons in the ventral hindlimb nerve and in forelimb nerves, a pattern that is virtually identical to the one previously observed in *Fz3*^*−/−*^ limbs. A comparison between the *Olig2-Cre;Rac1*^*CKO/−*^ and *Olig2-Cre;Fz3*^*CKO/−*^ phenotypes shows generally similar effects on cranial motor nerve axons and motor nuclei, but among different nuclei, the severity differs between the two genotypes. For example, a comparison of Fig. 2b and 2b’ in this work with Figure 1B and B’ of Hua et al. [17] shows a more severe loss of axons in the XIIth cranial nerve and adjacent C1 spinal nerve in the *Olig2-Cre;Rac1*^*CKO/−*^ embryo compared to the *Fz3*^*−/−*^ embryo.

Evidence for an interaction between PCP signaling—or, more generally, noncanonical Wnt signaling—and Rho family GTPases comes largely from studies of epithelial polarity in *Drosophila* and morphogenetic cell movements in *Xenopus* and zebrafish (reviewed in [59]). Early work based on dominant negative mutants and ectopic expression demonstrated a role for Rho family GTPases in establishing the cytoskeletal organization that underlies PCP-based polarity [60, 61] Later work, including experiments with loss-of-function alleles, implied that the relationship between PCP signaling and Rho GTPases may be indirect, with each representing a distinct pathway of information flow that converges on the cytoskeleton [8–10, 62]. Similar interpretations may apply to experiments with mammalian epithelia, in the context of directed cell movements and asymmetry in terminally differentiated cells [11, 63].

At present, data regarding the mechanism of Fz3/Celsr3 signaling in axon growth and guidance are limited. Aside from the experiments reported here, we are not aware of any experiments that have examined a possible relationship between Rho family GTPases and Fz3/Celsr3 function in axon guidance. Based on pharmacologic experiments in embryonic spinal cord explants, Frizzled-mediated guidance appears to be under the control of atypical protein kinase C zeta and phosphatidylinositol-3-kinase [64]. With respect to extrapolations from PCP mechanisms in epithelia, it is possible that axon guidance mechanisms are significantly different, since epithelial polarity depends on the core PCP proteins Vangl1 and Vangl2, whereas recent work suggests that axon guidance does not [19]. Additionally, the rapid extension and retraction of filopodia that accompany the exploratory movements of growth cones suggest that axon guidance may utilize PCP protein complexes that are more labile than the ones formed between epithelial cells. Interestingly, the work of Onishi et al. [65] has found a correlation between endocytic recycling of Fz3 at filopodial tips, Fz3 phosphorylation, and growth cone guidance. These data, together with the known role of Rho GTPases in regulating actin dynamics in growth cones [66], suggest that one potential mechanistic link between Rho family GTPases and Fz3 could be at the level of local regulation of actin polymerization/depolymerization in filopodia.

### Conclusions

The experiments reported here indicate a widespread requirement for *Rac1* in axon growth and guidance and a cell-autonomous defect in axon growth in *Rac1*^*−/−*^ motor neurons in culture. Following deletion of *Rac1* in the forebrain, thalamocortical axons were misrouted in a pattern that is indistinguishable from the pattern previously observed in *Frizzled3*^*−/−*^ and *Celsr3*^*−/−*^ forebrains. In the limbs, motor-neuron-specific deletion of *Rac1* produced a distinctive pattern of axon stalling that is virtually identical to the one previously observed in *Frizzled3*^*−/−*^ limbs. These similarities in axon growth and guidance phenotypes caused by *Rac1*, *Frizzled3*, and *Celsr3* loss-of-function mutations suggest a mechanistic connection between tissue polarity/planar cell polarity signaling and Rac1-dependent cytoskeletal regulation.

## Methods

### Mouse lines

The following mouse lines were used: *Brn3b*^*CKOAP*^ [35], *Foxg1-Cre* [67], *Hb9-GFP* [68], *Olig2-Cre* [23], *Pax6*α*-Cre* [29], *Rac1*^*CKO*^ [69], *Six3-Cre* [36], and *Wnt1-Cre* [70]. This study (protocol #M01M469) was approved by the Institutional Animal Care and Use Committee of the Johns Hopkins Medical Institutions, and mice were housed and handled in accordance with the approved Institutional Animal Care and Use Committee guidelines of the Johns Hopkins Medical Institutions.

### Alkaline phosphatase (AP) histochemistry

Throughout this work, the day of finding a copulation plug was counted as embryonic day (E)0.5, and the day when mice were born was counted as postnatal day (P)0. AP staining was performed on 200-μm-thick brain sections from P36 mice. Mice were anesthetized with ketamine/xylazine and then sacrificed by trans-cardiac perfusion with 4 % paraformaldehyde (PFA) [*w*/*v*, dissolved in phosphate buffered saline (PBS), pH 7.4], and brains were dissected out and further fixed in 4 % PFA overnight at 4 °C. Brains were embedded in 3 % low melting point agarose (*w*/*v*, dissolved in PBS) and sectioned at 200 μm on a vibratome. AP histochemistry was performed essentially as previously described [17, 18, 71]. Vibratome sections in PBS containing 2 mM MgCl_2_ were heated in a water bath at 69 °C for 90 min and then equilibrated in AP-staining buffer (0.1 M Tris, 0.1 M NaCl, 50 mM MgCl_2_, pH 9.5) overnight at room temperature. AP histochemistry was carried out in AP-staining buffer containing 0.34 μg/ml 4-nitro blue tetrazolium chloride (NBT) and 0.175 μg/ml 5-bromo-4-chloro-3-indolyl-phosphate (BCIP) (Roche Applied Science; Indianapolis, IN) for 8 h at room temperature with gentle horizontal shaking. Sections were dehydrated through an ethanol series and cleared with BBBA [2:1, benzyl benzoate (BB)/benzyl alcohol (BA)] (Sigma-Aldrich; St. Louis, MO) before imaging.

### Immunohistochemistry

The following primary antibodies were used: mouse anti-Brn3a (MAB1585; Millipore; Billerica, MA), rabbit anti-calbindin D28k (CB38; Swant; Marly, Switzerland), rabbit anti-calretinin (7699/4; Swant), rabbit anti-cleaved caspase3 (9661; Cell Signaling Technology; Danvers, MA), goat anti-choline acetyltransferase (ChAT) (AB144P; Millipore), rabbit anti-Foxp1 (ab16645; Abcam; Cambridge, England), mouse anti-glutamic acid decarboxylase [GAD-6; Developmental Studies Hybridoma Bank (DSHB); Iowa City, IA], rabbit anti-glial fibrillary acidic protein (GFAP) (RB-087-A; Thermo Scientific; Waltham, MA), rabbit anti-green fluorescent protein (GFP) (A11122; Life Technologies; Grand Island, NY), mouse anti-Islet1/2 (39.4D5; DSHB), rabbit anti-Islet1/2 (gift of Dr. Tom Jessell and Susan Morton, Columbia University), mouse anti-neurofilament (165 kDa, NF) (2H3; DSHB), goat anti-Sox10 (SC-17342; Santa Cruz Biotechnology; Dallas, TX), and rabbit anti-tyrosine hydroxylase (TH) (AB152; Millipore). Alexa Fluor 594-conjugated isolectin GS-IB4 (Life Technologies) was used to label blood vessels. Secondary antibodies were Alexa Fluor 488, 594, or 647 conjugated, donkey anti-goat, goat anti-mouse, or goat anti-rabbit IgG antibodies (Life Technologies).

Immunostaining was conducted as previously described [17, 18]. For retina cryosections, the enucleated eye was immersion fixed in 4 % PFA for 30 min at 4 °C, and then, the cornea and lens were carefully removed, and the eye cup was immersion fixed in 4 % PFA at 4 °C overnight, washed three times in PBS, equilibrated in 30 % sucrose (*w*/*v*, dissolved in PBS), embedded in optimal cutting temperature (OCT) compound (Sakura Finetek; Torrance, CA), frozen, and sectioned at 14 μm on a cryostat. For spinal cord cryosections, embryos were immersion fixed in 4 % PFA at 4 °C for 2 h, washed three times in PBS, equilibrated in 30 % sucrose, embedded in OCT compound, frozen, and sectioned at 14 μm on a cryostat. For vibratome sections, embryos or brains were immersion fixed in 4 % PFA at 4 °C overnight, washed three times in PBS, embedded in 3 % low melting point agarose, and sectioned at 120 μm on a vibratome. For whole-mount E11.5 embryos, and E12.5 and E13.5 limbs, samples were immersion fixed in 4 % PFA at 4 °C for 2 h and washed three times in PBS.

For immunostaining of 14-μm-thick cryosections and 120-μm-thick vibratome sections, sections were blocked in blocking solution [PBS containing 0.3 % Triton X-100 and 5 % normal goat serum (NGS) or normal donkey serum (NDS)] at room temperature for 1 h and incubated with primary antibody in blocking solution at 4 °C overnight. Sections were then washed five times in PBST (PBS containing 0.3 % Triton X-100) and incubated with secondary antibody in blocking solution at room temperature for 1 h (cryosections) or at 4 °C overnight (vibratome sections). Sections were then washed five times in PBST and mounted with Fluoromount-G (Southern Biotech; Birmingham, AL).

For immunostaining of whole-mount embryos and limbs, samples were first incubated in Dent’s Bleach [10 % H_2_O_2_, 13.3 % dimethyl sulfoxide (DMSO), 53.3 % methanol] at 4 °C for 24 h, washed with methanol five times, and fixed in Dent’s Fix (20 % DMSO, 80 % methanol) at 4 °C overnight. Samples were washed in PBS three times, incubated with primary antibody in blocking solution (20 % DMSO, 75 % PBST, 5 % NGS, 0.025 % sodium azide) at room temperature for 5–7 days with gentle end-over-end rotation, and then washed five times in PBST. Samples were incubated with secondary antibody in blocking solution at room temperature for 2 days with gentle end-over-end rotation and then washed five times in PBST. Samples were dehydrated in 50 % methanol/PBS and then 100 % methanol and cleared in BBBA.

### Dissociated spinal cord neuron cultures

Individual spinal cords from E11.5 embryos were dissected in Hank’s balanced salt solution (HBSS), and genotyping was performed on the remaining embryonic tissue. Spinal cords were diced, washed in HBSS, and incubated in 0.05 % trypsin-EDTA for 12 min at 37 °C, washed in DMEM with 10 % heat-inactivated normal horse serum, resuspended in the same media, triturated with a fire-polished Pasteur pipette, and then filtered through a 70-μm filter. Cells were plated on sterile glass coverslips coated in poly-D-lysine (20 μg/ml) and laminin (20 μg/ml) at a density of 50,000 cells/coverslip in 24-well plates. For immunohistochemistry, cells were washed twice in PBS with 0.5 mM MgCl_2_ and 1 mM CaCl_2_ and fixed for 10 min in 4 % PFA. GFP was visualized with chicken anti-GFP (GFP-1020; Aves Lab; Tigard, OR). Axon lengths were analyzed using the NeuronJ plug-in for ImageJ.

### Cholera toxin subunit B (CTB) anterograde tracing of optic tract axons

Adult mice were deeply anesthetized with ketamine/xylazine, and ~1–2 μl 2 mg/ml Alexa Fluor 594-conjugated cholera toxin subunit B (C-22842; Life Technologies) was intraocularly injected into the left eye of the mouse with a glass micro-needle. Four days later, mice were sacrificed by trans-cardiac perfusion, and brains were dissected out and further fixed in 4 % PFA overnight at 4 °C. Brains were embedded in 3 % low melting point agarose and sectioned at 100 μm on a vibratome. Sections were mounted on slides with Fluoromount-G and imaged by confocal microscopy.

### Microscopy and image analysis

Samples processed for AP histochemistry were imaged using a Zeiss Stemi V11 microscope with a color Axiocam CCD in combination with Openlab software. Immunostained samples were imaged using a Zeiss LSM700 confocal microscope with Zen software. Images of whole mounts were acquired with a 10× air objective at 10-μm intervals in the *Z* dimension, and the entire *Z* stack was either collapsed using maximum intensity projection or color-coded based on *Z*-dimension depth. BBBA-cleared embryos were positioned in custom-built metal holders consisting of a shallow triangular trough (sides: 2 cm × 2 cm × 1 cm; and depths: 1, 2, 3, or 4 mm). The trough was filled with BBBA and coverslipped during imaging.

### Quantification of motor and sensory neurons, measurement of nerve diameter, and statistical analysis

The number of neurons on each cryosection was manually quantified using ImageJ software. To measure the diameter of limb motor nerves, a line perpendicular to the nerve at the designated site was drawn in Adobe Illustrator and the width of the nerve along this line was then measured. Statistical comparisons were performed in Microsoft Excel.

## Abbreviations

AP: alkaline phosphatase
BBBA: benzyl benzoate and benzyl alcohol
CGE: caudal ganglionic eminence
CTB: cholera toxin subunit B
DRG: dorsal root ganglion
E: embryonic
Fz: frizzled
GCL: ganglion cell layer
GFAP: glial fibrillary acidic protein
HBSS: Hank’s balanced salt solution
HMC: hypaxial motor column
INL: inner nuclear layer
IPL: inner plexiform layer
LGE: lateral ganglionic eminence
LGN: lateral geniculate nucleus
LMC: lateral motor column
MGE: medial ganglionic eminence
MMC: medial motor column
MTN: medial terminal nucleus
NF: neurofilament
ONL: outer nuclear layer
OPN: olivary pretectal nucleus
P: postnatal
PA: pretectal area
PCP: planar cell polarity
PGC: preganglionic motor column
RGC: retinal ganglion cell
SC: superior colliculus
SCN: suprachiasmatic nucleus
WT: wild type
